# A Questionnaire-Derived Prediction Nomogram for Affected Semicircular Canal and Laterality of Benign Paroxysmal Positional Vertigo

**DOI:** 10.3390/diagnostics15192435

**Published:** 2025-09-24

**Authors:** Linlin Wang, Kaijun Xia, Yangming Leng, Renhong Zhou, Jingjing Liu, Hongchang Wang, Hongjun Xiao, Bo Liu

**Affiliations:** 1 Department of Otorhinolaryngology-Head Neck Surgery, ENT Institute, Union Hospital, Tongji Medical College, Huazhong University of Science and Technology, Wuhan 430022, China; 2020xh5020@hust.edu.cn (L.W.); xiakaijun626@hust.edu.cn (K.X.); lyangming@foxmail.com (Y.L.); zhourenhong@hust.edu.cn (R.Z.); 18971258151@189.cn (J.L.); 18696159860@163.com (H.W.); 2 Hubei Province Clinical Research Center for Deafness and Vertigo, Wuhan 430022, China

**Keywords:** benign paroxysmal positional vertigo, historical questionnaire, clinical prediction model

## Abstract

**Objective**: To investigate whether a detailed historical questionnaire can predict the affected semicircular canal and lateralization in patients with benign paroxysmal positional vertigo (BPPV). **Methods**: In this retrospective study, 459 patients with positional vertigo were evaluated, of whom 236 patients diagnosed with BPPV completed a symptom-based questionnaire. Based on the questionnaire data, logistic regression models were developed to predict lateralization and the affected semicircular canal. The clinical diagnosis of BPPV served as the reference standard. A nomogram was constructed based on the final logistic regression model, and model performance was evaluated using area under the receiver operating characteristic curve (AUC) in both training and validation cohorts. **Results**: Among 220 BPPV patients included, 152 (69.09%) were diagnosed with posterior semicircular canal BPPV (PSC-BPPV), 49 (22.27%) with horizontal semicircular canal canalolithiasis (HSC-BPPV-can), and 19 (8.64%) with horizontal semicircular canal cupulolithiasis (HSC-BPPV-cup). Waking up, lying down and rotating the head toward the left/right in the supine position, triggering vertigo, were significant predictors of the affected semicircular canal. Rotating the head toward the left/right in the supine position and vertigo duration were significantly predictors for lateralization. The AUCs were 0.787 and 0.714 for lateralization, and 0.814 and 0.842 for canal prediction in training and validation cohorts, respectively. **Conclusions**: The nomogram demonstrated good consistency with the reference standard diagnoses and may facilitate the identification of the affected side and canal in BPPV.

## 1. Introduction

Benign paroxysmal positional vertigo (BPPV) is a peripheral vestibular disorder characterized by recurrent transient dizziness and characteristic nystagmus induced by changes in head position relative to gravity [1]. The etiology of BPPV includes primary and secondary causes. Secondary causes include head trauma, vestibular neuritis, etc. [2,3,4]. In addition to positional maneuvers and differential diagnosis, history matters in the diagnosis and differential diagnosis of BPPV. Although diagnostic criteria for BPPV have been released [1,5,6,7], it is frequently not recognized by non-otolaryngologist physicians or some primary healthcare providers. van der Zaag-Loonen et al. reported that among individuals over 70 years of age who experienced dizziness and sought consultation from otolaryngology or other departments, the unrecognized BPPV cases was 13/226 (5.7%) [8]. Older patients with BPPV were reported to have higher risk of falls, depression and reduced activities of daily living scores [9]. The high proportion of unrecognized BPPV cases and its health care burden call for a convenient and effective tool for initial healthcare providers to predict the diagnosis of BPPV, e.g., a questionnaire. Previous studies found that the overall predictive capability of well-designed symptom questionnaires for dizziness patients achieved 70.3–84% [10,11,12].

Previous studies have shown that symptom-based questionnaires can effectively screen for BPPV [9,13,14]. These screening tools typically include characteristics such as vertigo/dizziness patterns, triggering factors, symptom duration, etc. Some Chinese researchers have developed rapid BPPV screening questionnaires with favorable reliability and validity metrics [15,16]. International studies have proposed questionnaires [17,18,19,20] and statistical models [21] for prediction of BPPV. However, some studies did not assign weighted scores to questions based on their importance, and patients’ response options were limited to binary (yes/no) options rather than graded options.

Some studies using questionnaire-based prediction models have focused on the differential diagnosis and subtype classification of BPPV. Jacobson et al. developed the 31-item Dizziness Symptom Profile (DSP) for differential diagnosis of common vestibular disorders including vestibular migraine, Ménière’s disease, BPPV, vestibular neuritis, superior semicircular canal dehiscence, and persistent postural-perceptual dizziness [12]. Shim et al. (2013) revealed that the provoking position (supine left and supine right) facilitated lateralization diagnosis in patients with posterior semicircular canal BPPV (PSC-BPPV) and geotropic horizontal semicircular canal BPPV (HSC-BPPV-geo) [22]. Kim et al. (2020) developed a 6-item questionnaire based on text mining of BPPV patients’ symptom descriptions, exhibit an overall accuracy in differential diagnosis, affected canal identification, lateralization diagnosis, and pathophysiological classification [23]. However, existing BPPV screening tools remain limited by excessive questions (some containing dozens of items), oversimplified binary responses, and insufficient statistical methods for optimal predictor selection.

Clinical prediction models, typically based on multivariable logistic regression, have emerged as effective tools for achieving accurate diagnosis and personalized treatment. These models enable clinicians to estimate health professionals to predict an individual’s risk of an outcome being present. This method has been used in studies of some inner ear disease, e.g., idiopathic sudden sensorineural hearing loss and Ménière’s disease [24,25]. However, no clinical prediction models have yet been applied to BPPV for identifying the affected side and canal. This study is the first to develop such models based on patient-reported clinical history obtained through a symptom-based questionnaire.

In this study, we aimed to develop clinical prediction models to investigate whether a detailed symptom-based questionnaire could predict lateralization, affected semicircular canal and pathophysiological type in BPPV patients.

## 2. Materials and Methods

### 2.1. Study Design and Population

This retrospective study was conducted at Wuhan Union Hospital and approved by its Ethics Committee. Data were collected from patients presenting with “positional dizziness/vertigo” between November 2022 and August 2023. All patients underwent Dix–Hallpike and Roll tests, and those diagnosed with posterior or horizontal semicircular canal BPPV based on diagnostic criteria proposed by the Bárány Society were eligible for this study [1]. Exclusion criteria were as follows: (1) Patients diagnosed with possible or probable BPPV according to Bárány classification [1]; (2) Patients diagnosed with multiple canal BPPV; (3) Patients whose positional vertigo was attributed to other vestibular or non-vestibular disorders, such as vestibular migraine, vestibular neuritis, sudden sensorineural hearing loss with vertigo, Meniere’s disease, head trauma, ototoxicity, etc.; (4) Patients with incomplete questionnaire data.

The eligible patients were randomly divided into a training cohort and a validation cohort at a ratio of 7:3 using R4.2.3, which ensured the outcome events were randomly distributed between the two groups. Data from the training cohort were used to develop the prediction model, while data from the validation cohort were used to independently evaluate its performance.

### 2.2. Questionnaire Information

Upon the patient’s initial visit, a detailed medical history was obtained through a structured questionnaire, investigating general demographic and clinical information. Demographic data included age and gender, while clinical information covered positions triggers of vertigo and symptom duration. Additionally, the patient’s past medical and family history were recorded.

Referring to previous studies, this study developed a scoring system to assess positions triggers of vertigo in BPPV patients (Table 1). This system included seven questions (Q1–Q7); Q1–Q6 each had three response options: ① None (the position does not trigger vertigo); ② Sometimes (the position triggers vertigo with moderate severity); ③ Often (the position triggers vertigo with significant severity). Furthermore, the self-reported duration of positional vertigo symptoms was categorized as follows: ① ≤10 s; ② 11–60 s; ③ >60 s.

### 2.3. Diagnostic Methods

The study included two primary outcome variables: BPPV subtype classification and affected ear laterality. BPPV subtypes were categorized as posterior semicircular canal BPPV (coded as 1) or horizontal semicircular canal BPPV (coded as 0). Affected ear laterality was coded as right ear (1) or left ear (0). Diagnoses of both BPPV subtype and affected ear were established using the Dix–Hallpike and Roll tests, conducted by two experienced specialists [5].

Predictive modeling was performed using data from the structured questionnaire, which included seven items assessing positional triggers of vertigo. Responses to these questionnaire items were converted into categorical variables based on predefined criteria and were used as potential predictors in the model. Independent predictors were identified through univariate analysis, multivariate analysis, and clinical judgment. The final predictive model was developed using logistic regression, incorporating significant questionnaire-based predictors along with demographic and clinical variables. The dataset was randomly divided into training (70%) and validation (30%) cohorts.

### 2.4. Statistical Analysis

Data were analyzed using SPSS 26.0 software. Quantitative data were first assessed for normality and homogeneity of variance. Normally distributed data were presented as mean ± standard deviation ($\bar{x}$ ± s), while non-normally distributed data were expressed as the median (interquartile range, IQR). Group comparisons for normally distributed data were conducted using an independent samples *t*-test, whereas non-normally distributed data were analyzed using rank-sum tests. Categorical variables were expressed as frequencies and percentages, and between-group comparisons were performed using the chi-square test. *p* value < 0.05 was considered statistically significant.

Predicative modeling and nomogram development were conducted using R4.2.3, including pROC and rms packages. Model performance was evaluated using the receiver operating characteristic (ROC) curve and area under the curve (AUC), with an AUC > 0.7 indicating good discriminative ability. Calibration was evaluated using calibration curves to assess concordance between predicted probabilities and observed outcomes.

## 3. Results

### 3.1. General Characteristics

Initially, 459 patients presenting with positional vertigo were invited to complete the questionnaire. After excluding 223 individuals who did not meet the inclusion criteria, 14 with incomplete data, and 2 with multiple semicircular canal involvement, 220 patients were ultimately included in the final analysis. The sample size satisfies the minimum requirement for the predictive model according to the 10 EPV guidelines [26].

Among the 220 patients, 78 (35.45%) were male and 142 (64.55%) were female, with a mean age of 51.99 ± 13.42 years. According to the BPPV diagnostic criteria, 152 patients (69.09%) were diagnosed with PSC-BPPV and 68 patients (30.91%) were diagnosed with horizontal semicircular canal BPPV (HSC-BPPV). Of all cases, 197 (89.54%) were classified as canalolithiasis and 23 (10.45%) as cupulolithiasis. Regarding laterality, 90 (41%) were left-sided and 130 (59%) right-sided.

The training cohort consisted of 154 cases, and the validation cohort consisted of 66 cases. No significant differences in age, sex, or affected side were observed between the two groups (Table 2).

### 3.2. Predictive Factors

Univariate and multivariate logistic regression analysis identified four independent predictors of affected semicircular canal in BPPV patients. These predictors included waking up (*p* = 0.007; OR = 3.27, 95% CI: 1.39–7.70), lying down (*p* = 0.053; OR = 2.47, 95% CI: 0.99–6.16), rotating the head toward the left in the supine position (*p* < 0.001; OR = 0.15, 95% CI: 0.05–0.45), and rotating the head toward the right in the supine position (*p* = 0.007; OR = 0.22, 95% CI: 0.07–0.66) (Table 3).

Multivariate logistic regression identified three independent predictors of affected-side laterality in BPPV: rotating the head toward the left in the supine position (*p* < 0.001; OR = 0.07, 95% CI: 0.02–0.21), rotating the head toward the right in the supine position (*p* < 0.001; OR = 5.13, 95% CI: 2.27–11.57), and vertigo duration (*p* = 0.041; OR= 2.15, 95% CI: 1.03–4.49) (Table 4).

The univariate and multivariate logistic regression analysis identified that age (*p* = 0.017; OR = 0.99, 95% CI: 0.98–0.99), disease course (*p* = 0.025; OR = 0.33, 95% CI: 0.13–0.87), lying down (*p* = 0.003; OR = 4.44, 95% CI: 1.65–11.94), and vertigo duration (*p* = 0.007; OR = 0.14, 95% CI: 0.03–0.58) were independent predictors of pathophysiological type in BPPV patients (Table 5).

### 3.3. Prediction Model Construction and Assessment

**Figure 1:**
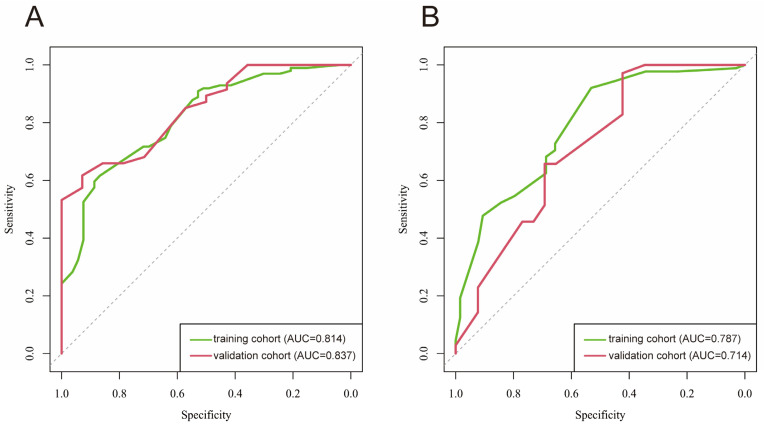
ROC graphs of the affected semicircular canal prediction model (**A**) and the affected side prediction model (**B**). Green curve: training cohort; red curve: validation cohort; AUC: area under the curve.

The affected semicircular canal and affected side prediction model were built on the basis of the results of the multivariate logistic regression analysis. For the affected semicircular canal prediction model, the areas under the receiver operating characteristic curve (AUC) in the training cohort model and the validation cohort model were 0.814 and 0.837. Both AUCs exceed 0.7, and the AUC of the validation cohort is even better than the training cohort, indicating that the model has good discrimination (Figure 1A). For the affected side prediction model, the AUCs in the training cohort model and the validation cohort model were 0.787 and 0.714. These results also suggest good discriminatory ability (Figure 1B).

### 3.4. Nomogram and Calibration Curve Preparation

**Figure 2:**
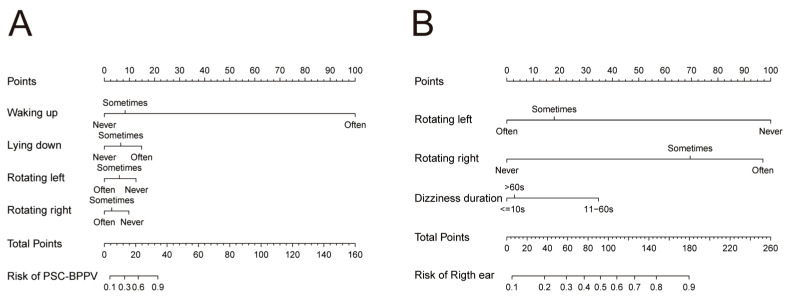
Nomogram models for predicting the posterior semicircular canal-affected (**A**) and right-affected (**B**) in patients with BPPV. PSC-BPPV: posterior semicircular canal benign paroxysmal positional vertigo.

Combined with clinical experience and the results of univariate and multivariate analyses, waking up, lying down, rotating the head toward the left in the supine position, and rotating the head toward the right in the supine position were selected as predictors of the nomogram clinical prediction model for affected semicircular canal in BPPV patients (Figure 2A). Additionally, rotating the head toward the left/right in the supine position and vertigo duration were incorporated into the nomogram model for predicting the affected side (Figure 2B).

**Figure 3:**
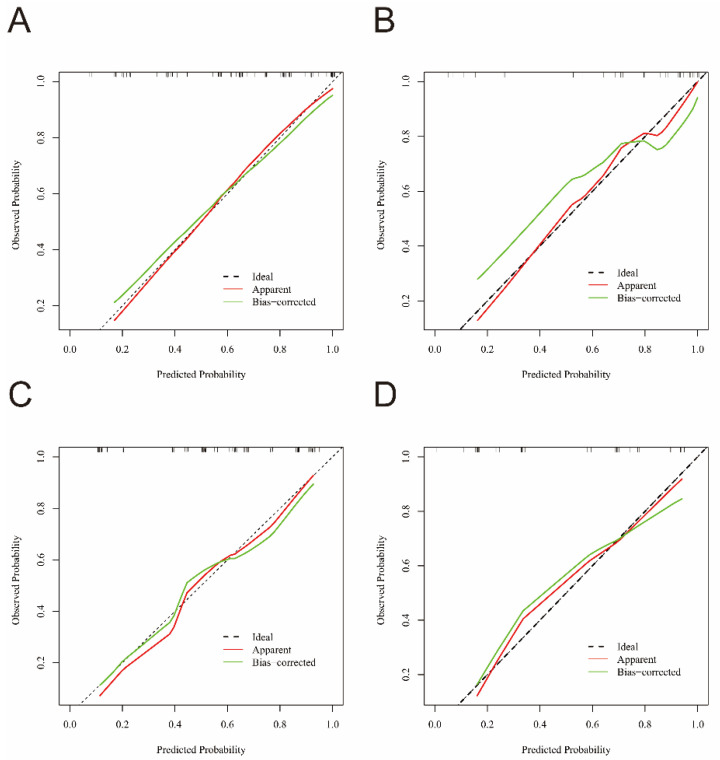
Calibration curve of the affected semicircular canal (**A**) and affected side (**B**) in training group. Calibration curve of the affected semicircular canal (**C**) and affected side (**D**) in validation group.

The calibration curves for the nomograms predicting BPPV the affected semicircular canal and affected side are shown in Figure 3. Both the apparent and bias-corrected calibration curves closely approximate the ideal calibration line for both the training and validation cohorts. This finding indicates good agreement between the predicted probabilities and observed outcomes, demonstrating the strong calibration capability of our nomogram.

### 3.5. Web-Based Nomogram

To facilitate clinical application and individualized risk prediction, we developed two web-based nomograms using Shiny for R4.2.3, available at https://bppvprediction.shinyapps.io/bppvprediction1app/ (accessed on 30 April 2025) and https://bppvprediction.shinyapps.io/bppvprediction2app/ (accessed on 30 April 2025). These interactive tools enable clinicians to input key clinical parameters to generate estimated probabilities of the affected semicircular canal and affected ear. The first tool (App 1) is designed to predict the affected semicircular canal, while the second tool (App 2) predicts the affected side. In both tools, clinicians can enter the patient’s questionnaire responses into drop-down menus. The tools then provide predicted probabilities with 95% CIs, displayed numerically and graphically. These nomograms allow clinicians to visualize and quantify individualized risk predictions in real-time, supporting evidence-based clinical decision-making and enhancing patient counseling.

## 4. Discussion

Dizziness/vertigo is one of the most common chief complaints encountered in clinical practice, particularly among patients presenting to neurology and otolaryngology clinics. The diagnosis and differential diagnosis of inner ear disease mainly rely on medical history investigation [27]. BPPV is the most prevalent peripheral vestibular disorder, characterized by positional vertigo triggered during daily activities such as waking up, lying down, or rolling over [28]. Based on the questionnaire results, we found the independent predictors of the affected semicircular canal, the affected side and pathophysiological type of BPPV.

### 4.1. Two Clinical Prediction Models of BPPV

Our studies employed logistic regression to filter the independent predictors and develop two clinical prediction models for affected semicircular canal and affected side of BPPV. Previous studies on BPPV prediction employed various statistical methods to identify predictors, including principal components analysis [12], variance analysis [17], consistency analysis [22,23], etc. The logistic regression model employed in our study enables adjustment for confounding factors, assessment of interaction effects, and quantification of effect magnitudes. Our study is the first to establish nomogram models predicting the affected semicircular canal and side in BPPV. Importantly, the questionnaire-based predictive model may have particular value in primary healthcare settings, where clinicians are often non-specialists and advanced vestibular testing such as video nystagmography (VNG) used for capturing nystagmus may be unavailable. Patients with BPPV may undergo unnecessary diagnostic testing prior to referral to a specialist [29,30]. In such cases, our models could facilitate early diagnosis and timely management, thus reducing the financial burden and fall risk of these patients.

### 4.2. The Independent Predictors for Affected Semicircular of BPPV

This study found that vertigo-provoking positions, i.e., waking up, lying down, and rotating the head toward the left/right in the supine position (Q1–Q4), serve as independent predictors for identifying the affected semicircular canal in BPPV (HSC-BPPV/PSC-BPPV). In the study by Kim et al., regarding the question “ Which positional change makes you feel more dizzy? (1) Lying down or getting out of bed? (2) Turning your head (or body) while lying down?”, 102 (86.4%) patients with PSC-BPPV chose the former, while 30 (71.4%) patients with HSC-BPPV selected the latter. This question demonstrated an accuracy of 82.5% in distinguishing posterior semicircular canal BPPV from horizontal semicircular canal BPPV [23]. The results of the present study were consistent with the above study, suggesting the predictive value of provoking position in identifying the affected semicircular canal in BPPV. However, the study by van Dam et al. showed no significant differences in vertigo-provoking position (e.g., lying down, rolling over, bending, or getting up) between patients with PSC-BPPV and HSC-BPPV [18]. The discrepancy between their findings and ours may result from differences in questionnaire design. Their study used binary options (yes/no), whereas ours employed graded options (none/sometimes/often), which may enhance the predictive capability of our prediction models. Shim et al. retrospectively analyzed the results of questionnaire from patients with horizontal and posterior semicircular canal BPPV, finding that “waking up” was the most common vertigo-provoking position in both horizontal (45.1%) and posterior (61.5%) canal BPPV patients, without significant difference [22]. The study of Shim et al. required participants to select one or more vertigo-provoking positions from a list of 10 items. Differences in questionnaire structure may account for the inconsistency between the two studies.

From an anatomic view, head movements during lying down or waking up are close to the anatomical plane of the posterior semicircular canal, facilitating gravity-dependent displacement of debris, thereby triggering vestibular symptoms for patients with posterior semicircular canal BPPV [31]. For patients with horizontal semicircular canal BPPV, the plane of head rotating in the supine position is close to the anatomical plane of the horizontal semicircular canal. This spatial orientation facilitates gravity-dependent migration of debris of patients with horizontal semicircular canal BPPV, ultimately provoking vestibular symptoms.

### 4.3. The Independent Predictors for Affected Side of BPPV

This study found that rotating the head toward the left/right in the supine position provoking vertigo (Q3 and Q4) helps in predicting the affected side of PSC-BPPV and HSC-BPPV. Shim et al. analyzed questionnaire responses from BPPV patients and demonstrated that vertigo provoked by rotating the head toward left/right in the supine position could predict the affected side in PSC-BPPV and HSC-BPPV-geo but not in apogeotropic horizontal semicircular canal BPPV (HSC-BPPV-apo) [22]. In the current study, questionnaire results from HSC-BPPV-apo and HSC-BPPV-geo patients were not separated, which may explain the partial inconsistency. Additionally, our study employed univariate and multivariate logistic regression to filter independent predictors, methodological differences may contribute to the discrepancies.

Our study indicated that rotating the head toward the left/right in the supine position provoking vertigo (Q3 and Q4) remained an independent predictor of the affected side in PSC-BPPV, although the plane of head movement in Q3/Q4 is not fully parallel with the anatomical plane of the posterior semicircular canal. Shim et al. reported that among 239 PSC-BPPV patients, the proportions of provoking positions—waking up, lying down, rotating the head toward the right in the supine position, and rotating the head toward the left in the supine position—were 61.5%, 45.6%, 26.4%, and 22.6%, respectively [22]. In Kim et al.’s study, 13.6% of PSC-BPPV patients experienced vertigo when rotating the head toward the left/right in the supine position [23]. These findings, along with our results, suggest that for PSC-BPPV patients, although less frequently, rotating the head in the supine position may also induce displacement of debris, thereby provoking vestibular symptoms.

For PSC-BPPV patients experiencing vertigo during rotating the head in the supine position, previous studies have proposed several hypotheses. Ichijo et al. classified PSC-BPPV into four subtypes based on nystagmus patterns during the new roll test: supine type, lateral type, head-hanging type, and Dix–Hallpike type. For the lateral type, no nystagmus is observed after reclining from sitting position to the supine position, but transient vertical/torsional nystagmus appears when the head is rotated 45° to the affected side [32]. The authors hypothesized that in this type, the debris is in the ampulla in the supine position, and enters the long arm and sinks under gravity when the head is rotated 45° toward the affected side. Cui et al. observed vertical nystagmus during the roll test in 74/175 (42.3%) PSC-BPPV patients [33]. Taking left PSC-BPPV as an example, in supine position, Cui et al. considered that the debris is at the lowest point of the long arm of the canal. When the head is rotated 90° to the right side, the long arm becomes positioned at the highest elevation, and the debris move toward the crus commune, eliciting vertical torsional nystagmus. These findings, including ours, suggest that variations in the anatomical location of otoliths may underlie differences in vertigo-triggering maneuvers among PSC-BPPV patients.

In the present study, patients with vertigo lasting 11–60 s were more likely to have right-sided BPPV compared to those with episodes ≤ 10 s. Previous studies have not identified a relationship between vertigo episode duration and the laterality of BPPV. Our cohort exhibited an imbalanced distribution of symptom duration: 65% (143/220) of participants reported episodes ≤ 10 s, whereas only 28.2% (62/220) experienced episodes of 11–60 s. This disproportionate proportion of the 11–60 s subgroup may raise bias in statistical analyses. Our duration grouping was based on clinical observation, since most patients described their vertigo or dizziness as lasting only a few seconds, whereas others reported episodes that persisted for several tens of seconds but typically less than one minute. It is possible that refining this classification system for vertigo duration might yield different results. Notably, the accuracy of self-reported vertigo duration may be affected by the time interval between symptom onset and clinical assessment. The longer the delay before seeking medical care, the greater the potential for recall bias. In addition, BPPV symptoms often remit or diminish over time, potentially leading patients to underestimate the severity or duration of their episodes at the time of evaluation [34]. Although significant correlation was found between vertigo episode and the laterality of BPPV, future studies incorporating objective duration measurement and refined classification system are needed to validate these preliminary findings.

### 4.4. The Independent Predictor of Pathological Type of BPPV

In our present study, the episode duration of vertigo was a predictive factor for the pathophysiological type of BPPV (canalithiasis/cupulolithiasis), which is consistent with our expectations. For PSC-BPPV, characteristic nystagmus lasting < 1 min during the Dix–Hallpike test indicates posterior canal canalithiasis, whereas characteristic nystagmus persisting > 1 min suggests posterior canal cupulolithiasis. For horizontal semicircular canal BPPV, characteristic nystagmus lasting < 1 min during the Roll test corresponds to horizontal canal canalithiasis, while persistent geotropic nystagmus >1 min indicates horizontal canal cupulolithiasis [1,35]. To date, there have been no predictive studies differentiating between BPPV canalithiasis and BPPV cupulolithiasis based on history-based investigations.

Among the 220 patients included, only 23 were diagnosed with the cupulolithiasis type. Due to the small sample size and the potential risk of model overfitting and biased estimates, we chose not to develop a predictive model for pathophysiological classification. Future studies with larger and less biased samples are needed to explore predictive modeling in this context.

### 4.5. Strengthens and Limitations

This study is, to our knowledge, the first to use logistic regression modeling to predict the affected side and semicircular canal subtype of BPPV using a symptom-based questionnaire. By integrating questionnaire-derived data with logistic regression analysis, we developed two clinical prediction models capable of estimating individualized probabilities for specific BPPV subtypes. Compared to traditional predictive studies, our models export the probability of a certain subtype of BPPV and showed good agreement between the predicted diagnoses and gold standard diagnoses. These models may assist primary healthcare providers when evaluating suspected BPPV cases.

This study also has certain limitations. First, the analysis excluded ASC-BPPV and multi-canal BPPV cases. Although the prevalence of ASC-BPPV (1.0–2%) and multi-canal BPPV (4.7–12.2%) varies across studies [36,37,38,39], ASC-BPPV and multi-canal BPPV were commonly recognized as rare cases [1,40]. The pathophysiology of these subtypes remains poorly understood [41], and their potential association with secondary causes (e.g., head trauma in multi-canal BPPV) introduces confounding factors [36,42]. By excluding ASC-BPPV, multi-canal BPPV cases and secondary cases, we prioritized investigating the natural course of primary BPPV to strengthen result validity. Currently, no studies have established clinical prediction models for ASC-BPPV and multi-canal BPPV. Second, this study included only patients with confirmed BPPV. Future research should expand to differentiate BPPV from other inner ear disorders, such as Ménière’s disease and vestibular migraine. Third, the time interval between symptom onset and clinical evaluation varied among patients. Delays in seeking medical care delays may lead to recall bias, and spontaneous symptom remission over time may further alter patients’ perception of symptom severity or duration. Fourthly, as the focus of this study was to establish a rapid screening tool for BPPV, we developed this questionnaire-derived prediction model especially for those primary healthcare providers who do not have access to advanced vestibular testing for recording and analyzing nystagmus. Future study incorporating history questionnaire and nystagmus characteristics using VNG or portable nystagmus recording device will be warranted.

## 5. Conclusions

Vertigo triggered by turning the head to the left/right in the supine position and episode duration showed high predictive value for BPPV lateralization. Vertigo induced by waking up, lying down, and turning the head to the left/right in the supine position exhibited strong predictive utility for identifying HSC-BPPV and PSC-BPPV. Our predictive models, which can identify the affected side and affected semicircular canal in BPPV, showed good agreement between the predicted diagnoses and gold standard diagnoses.

## Figures and Tables

**Table 1:** The questionnaire used in our present study.

Questions
Q1: Do you feel vertigo when you wake up?
Q2: Do you feel vertigo when you lie down?
Q3: Do you feel vertigo when you lean or look upward?
Q4: Do you feel vertigo when you bend or look downward?
Q5: Do you feel vertigo when rotating the head toward the left in the supine position?
Q6: Do you feel vertigo when rotating the head toward the right in the supine position? Q7: How long does the vertigo last?

**Table 2:** Demographic characteristics and clinical profile of patients in the training cohort and validation cohort.

Variables	Training Cohort (*n* = 154)	Validation Cohort (*n* = 66)	*p*
Age, mean ± SD	51.84 ± 13.99	52.33 ± 12.06	0.805
Disease course, medium (Q1, Q3)	16.50 (7.00, 30.00)	14.00 (8.00, 33.75)	0.960
Gender, *n* (%)			0.268
Female	103 (66.88)	39 (59.09)	
Male	51 (33.12)	27 (40.91)	
Waking up, *n* (%)			0.401
Never	29 (18.83)	15 (22.73)	
Sometimes	103 (66.88)	38 (57.58)	
Often	22 (14.29)	13 (19.70)	
Lying down, *n* (%)			0.501
Never	31 (20.13)	14 (21.21)	
Sometimes	100 (64.94)	46 (69.70)	
Often	23 (14.94)	6 (9.09)	
Leaning or looking upward, *n* (%)			0.618
Never	82 (53.25)	37 (56.92)	
Sometimes	72 (46.75)	28 (43.08)	
Bending or looking downward, *n* (%)			0.426
Never	81 (52.60)	38 (58.46)	
Sometimes	73 (47.40)	27 (41.54)	
Rotating the head toward the left in the supine position, *n* (%)			0.182
Never	57 (37.01)	16 (24.24)	
Sometimes	77 (50.00)	40 (60.61)	
Often	20 (12.99)	10 (15.15)	
Rotating the head toward the right in the supine position, *n* (%)			0.146
Never	41 (26.62)	18 (27.27)	
Sometimes	80 (51.95)	41 (62.12)	
Often	33 (21.43)	7 (10.61)	
Vertigo duration, *n* (%)			0.955
<10 s	100 (64.94)	43 (65.15)	
10 s~60 s	43 (27.92)	19 (28.79)	
>60 s	11 (7.14)	4 (6.06)	

*n*: number of cases.

**Table 3:** Results of the univariate and multivariate logistic regression analyses of clinical indicators associated with affected semicircular canal involvement in BPPV.

Variables	Univariate Logistic Regression		Multivariate Logistic Regression	
	OR (95%CI)	*p*	OR (95%CI)	*p*
Age	0.99 (0.97~1.01)	0.429		
Disease course	1.00 (0.99~1.01)	0.477		
Gender	0.58 (0.32~1.05)	0.074	0.61 (0.30~1.26)	0.18
Waking up	4.42 (2.16~9.03)	<0.001	3.27 (1.39~7.70)	0.007
Lying down	3.20 (1.61~6.38)	<0.001	2.47 (0.99~6.16)	0.053
Leaning or looking upward	0.89 (0.50~1.58)	0.679		
Bending or looking downward	0.63 (0.35~1.12)	0.113		
Rotating the head toward the left in the supine position	0.14 (0.05~0.36)	<0.001	0.15 (0.05~0.45)	<0.001
Rotating the head toward the right in the supine position	0.23 (0.09~0.56)	0.001	0.22 (0.07~0.66)	0.007
Vertigo duration	0.60 (0.20~1.80)	0.365		

**Table 4:** Results of the univariate and multivariate logistic regression analyses of clinical indicators associated with affected-side laterality in BPPV.

Variables	Univariate Logistic Regression		Multivariate Logistic Regression	
	OR (95%CI)	*p*	OR (95%CI)	*p*
Age	1.00 (0.98~1.02)	0.828		
Disease course	1.00 (0.99~1.00)	0.359		
Gender	0.61 (0.35~1.06)	0.082	0.58 (0.30~1.13)	0.108
Waking up	1.69 (0.68~4.18)	0.255		
Lying down	1.52 (0.58~3.99)	0.395		
Leaning or looking upward	1.17 (0.68~2.02)	0.563		
Bending or looking downward	1.27 (0.74~2.18)	0.394		
Rotating the head toward the left in the supine position	0.11 (0.04~0.29)	<0.001	0.07 (0.02~0.21)	<0.001
Rotating the head toward the right in the supine position	2.94 (1.54~5.61)	0.001	5.13 (2.27~11.57)	<0.001
Vertigo duration	2.21 (1.15~4.22)	0.017	2.15 (1.03~4.49)	0.041

**Table 5:** Results of the univariate and multivariate logistic regression analyses of clinical indicators associated with pathophysiological type in BPPV.

Variables	Univariate Logistic Regression		Multivariate Logistic Regression	
	OR (95%CI)	*p*	OR (95%CI)	*p*
Age	0.99 (0.98~0.99)	0.01	0.99 (0.98~0.99)	0.017
Disease course	0.35 (0.15~0.82)	0.016	0.33 (0.13~0.87)	0.025
Gender	1.08 (0.40~2.91)	0.881		
Waking up	1.08 (0.40~2.91)	0.881		
Lying down	3.31 (1.36~8.04)	0.008	4.44 (1.65~11.94)	0.003
Leaning or looking upward	1.10 (0.46~2.64)	0.824		
Bending or looking downward	1.10 (0.46~2.64)	0.824		
Rotating left	1.36 (0.53~3.45)	0.524		
Rotating right	0.60 (0.19~1.94)	0.397		
Vertigo duration	0.20 (0.06~0.67)	0.009	0.14 (0.03~0.58)	0.007

## Data Availability

The data supporting the findings of this study are available from the corresponding author on reasonable request.

## Abbreviations

The following abbreviations are used in this manuscript:

BPPV	Benign paroxysmal positional vertigo
DSP	Dizziness Symptom Profile
PSC-BPPV	Posterior semicircular canal benign paroxysmal positional vertigo
HSC-BPPV	Horizontal semicircular canal benign paroxysmal positional vertigo
HSC-BPPV-geo	Geotropic horizontal semicircular canal benign paroxysmal positional vertigo
HSC-BPPV-apo	Apogeotropic horizontal semicircular canal benign paroxysmal positional vertigo
ASC-BPPV	Anterior semicircular canal benign paroxysmal positional vertigo
HSC-BPPV-can	horizontal semicircular canal canalolithiasis
HSC-BPPV-cup	horizontal semicircular canal cupulolithiasis
CI	confidence interval
IQR	interquartile range
ROC	receiver operating characteristic
AUC	Area under the receiver operating characteristic curve
VNG	video nystagmography
